# Assessing the accuracy of routinely collected data and their potential use in pressure ulcer trials

**DOI:** 10.1186/1745-6215-16-S2-P39

**Published:** 2015-11-16

**Authors:** Isabelle Smith, Sarah Brown, Susanne Coleman, Lyn Wilson, Jane Nixon

**Affiliations:** 1 Leeds Institute of Clinical Trials Research, Leeds, UK

## 

Pressure ulcer trials often use development of category≥2 pressure ulcers as an endpoint[1]. Research nurses regularly assess patient's skin to capture pressure ulcer development, however missed assessments may lead to missed pressure ulcers and capturing data from routine care may be an alternative.

Pressure ulcer monitoring systems have been introduced in the English NHS including the Safety Thermometer. A project, funded by the Tissue Viability Society and supported by NHS England, comprised a pressure ulcer/wound audit to assess the accuracy of current monitoring systems. The pressure ulcer/wound audit was conducted on the Safety Thermometer census date (October 2014) in a stratified random sample of Trusts providing in-patient services in England, using ‘gold-standard’ prevalence methods[2]. In addition the Trust audit lead completed a questionnaire to elicit information about local practice for pressure ulcer reporting.

The results show low accuracy of routine pressure ulcer data sources; the weighted sensitivity estimate(95% CI) for the safety thermometer was 48.2%(35.4%-56.7%). The pressure ulcer/wound audit indicated that pressure ulcers reported on monitoring systems may not be readily obtained from clinical notes. These results support published data comparing ward and research nurse data[1]. Questionnaire responses indicated there was variation across Trusts in terms of pressure ulcer reporting across all monitoring systems. Therefore, results of the pressure ulcer/wound audit and questionnaire indicate that current routine data sources for pressure ulcer monitoring would not be satisfactory for use in pressure ulcer trials.
